# Comparative Analysis of Mechanical and Morphological Properties of Cordenka and Ramie Fiber-Reinforced Polypropylene Composites

**DOI:** 10.3390/ma17225519

**Published:** 2024-11-12

**Authors:** Wycliffe Ondiek, Amirul Ridzuan, Masaki Iwamoto, Arnaud Macadre, Koichi Goda

**Affiliations:** 1 Department of Industrial & Energy Engineering (IEEN), Egerton University, Egerton-Njoro P.O. Box 536-20115, Kenya; wycliffe.ondiek@egerton.ac.ke; 2 Graduate School of Sciences and Technology for Innovation, Yamaguchi University, Ube 755-8611, Japanb009vd@yamaguchi-u.ac.jp (M.I.); 3 Department of Mechanical Engineering, Yamaguchi University, Ube 755-8611, Japan; macadre@yamaguchi-u.ac.jp

**Keywords:** green composites, wood plastic composites, cellulose nanofibers, artificial cellulosic fibers, M-PaRI, injection molding, tensile test, impact test

## Abstract

The depletion of conventional materials and their adverse environmental impacts have prompted a shift toward sustainable alternatives in composite materials engineering. In pursuit of this objective, this study investigated the mechanical properties of polypropylene matrix composites reinforced with Cordenka, an artificial cellulose fiber, and compared them to those reinforced with ramie, a natural cellulose fiber. Continuous strand composites were developed using the Multi-Pin-assisted Resin Infiltration (M-PaRI) process. The strands were subsequently sectioned into 15 mm lengths and injection-molded into dumbbell and strip specimens for mechanical characterization. The results showed that 20 wt% Cordenka/PP composites exhibited a tensile strength of 68.7 MPa, 2.04 times higher than neat PP and 1.66 times greater than the 20 wt% ramie/PP composites. Impact testing further demonstrated that Cordenka/PP composites absorbed 2 to 2.5 times more impact energy than ramie/PP composites, regardless of the presence of notches. Fiber length analysis indicated that Cordenka fibers maintained their length beyond the critical fiber length, allowing for efficient stress transfer and acting as a more effective reinforcement compared to ramie fibers, which were below this threshold. Consequently, the Cordenka/PP composites exhibited significantly enhanced mechanical performance. Scanning electron microscopy (SEM) analysis revealed fewer fiber pullouts in ramie-reinforced composites, suggesting superior interfacial adhesion to the PP matrix, although it did not translate to higher mechanical properties. These findings underscore the potential of Cordenka as a sustainable alternative to synthetic, non-biodegradable fibers in PP composites, providing improved mechanical properties and promising prospects for advanced composite applications.

## 1. Introduction

Green plastics, commonly referred to as biodegradable plastics, are materials that undergo decomposition into water and carbon dioxide through the activity of microorganisms and enzymes present in natural environments [1,2]. As a result, green composites can integrate not only natural fibers, which are frequently utilized as reinforcement, but also biodegradable fibers, enhancing their sustainability and environmental impact. Since regenerated cellulose fibers such as rayon, cupro, and lyocell, and biomass-derived fibers such as polylactic acid (PLA) fibers exhibit biodegradability, composite materials that use these fibers as reinforcing materials also fall under the category of green composites (hereinafter abbreviated as GCs). Furthermore, in a broader sense, the term ’green’ can also include recyclable materials, allowing composites with thermoplastic resins to be considered GCs. Thermoplastic resins include polypropylene (PP), polyethylene (PE), polystyrene (PS), and polyvinyl chloride (PVC). These resins, known as the ‘big four general-purpose resins’, are easily accessible due to their low cost and high production volume, enabling their use in the manufacture of affordable green composites.

The mechanical properties of the aforementioned resins are summarized in Table 1 [3,4,5]. As shown in the table, PP has the lowest specific gravity among the four major resins, ranging from 0.90 to 0.91, making it the lightest option available. Furthermore, PP exhibits excellent processability, toughness and high flexural strength, alongside impact resistance and rigidity, particularly when considering its strength-to-weight ratio. Its high surface hardness provides excellent scratch resistance and contributes to its glossy finish. PP also possesses a high melting point of 160–170 °C and demonstrates outstanding resistance to chemicals, oils, and stress cracking. Composed solely of carbon and hydrogen, the incineration of PP produces minimal harmful gases, such as chlorine, which implies a lower environmental impact.

Among the eco-friendly reinforcement materials currently under extensive investigation, cellulose nanofibers (CNFs) extracted from the microfibrils of woody or herbaceous plants using physical or chemical methods stand out. Many researchers have developed composite materials by combining CNF with various resins [6,7,8,9,10,11,12,13,14]. The extraction of CNF represents one method for processing natural fibers. In addition to this method, natural fibers also have a well-established history in the textile industry, where they undergo a process known as ’regeneration’, applicable to both woody and herbaceous fibers. Until now, there has not been as much focus on regenerated fibers as there is on green composites. If regenerated fiber-reinforced composites can demonstrate mechanical properties comparable to CNF-based composites, they could be effectively utilized in modern material production and structural applications, particularly due to their biodegradability, similar to natural fiber composites. Therefore, this study focused on evaluating the mechanical properties of GCs reinforced with regenerated fiber and thermoplastic resin as the matrix material.

The regenerated fiber examined in this study is Cordenka fiber, which, although not classified as a natural fiber, contains cellulose. It falls within the category of regenerated cellulose fibers. Cordenka fibers are traditionally used in tire cord applications, but recent studies have explored their properties when compounded with matrices such as PLA, polypropylene (PP), and epoxy resin (EP). These studies have compared the properties with those of typical GCs, i.e., composites containing cellulose-based natural fibers such as flax and jute. Bax et al. [15] compared Cordenka/PLA injection molded materials with Flax/PLA counterparts, reporting that the former exhibited superior tensile and impact strength. Ganster et al. [16] compared Cordenka/PP injection molded materials with glass fiber/PP counterparts, noting that Cordenka/PP materials showed higher tensile strength and significantly improved impact strength in unnotched specimens. Such improvements are attributed to the high toughness of Cordenka fibers.

Kovalovs et al. [17] analyzed the impact response of hybrid biocomposites made from polypropylene homopolymer (HP400R) reinforced with Cordenka fibers and softwood microfibers (Weho 500). Their findings demonstrated that combining these fillers improved the impact properties, with the incorporation of Cordenka fibers increasing the absorbed energy by 372% compared to unreinforced polypropylene and by 243% compared to polypropylene with 30 wt% Weho fibers. Similarly, Meredith et al. [18] created laminates by combining Biotex flax, Lineo flax, Lineo flax preg, and Cordenka rayon with MTM49, MTM28, and Araldite LY5150 high-strength epoxy resins, and conducted various tests to determine their mechanical properties. Their results showed that Cordenka MTM49 and Biotex MTM49 composites exhibited the highest compressive strengths of 299.6 MPa and 223.5 MPa, respectively. Additionally, Cordenka composites registered a higher tensile strength of 92.6 MPa compared to the flax materials, which ranged from 63 to 77.6 MPa.

Malaba et al. [19] examined the mechanical properties of unidirectional Cordenka fiber-reinforced Furan resin biocomposites with varying high fiber weight fractions. They found that increasing the fiber weight fraction from 51 wt% to 64 wt% enhanced the tensile strength of the biocomposite. However, further increasing the fiber content to 75 wt% led to a reduction in tensile strength. Scanning electron microscopy (SEM) analysis of the tensile fracture surfaces revealed a decline in fiber–matrix adhesion as fiber content increased. This deterioration was primarily attributed to diminished fiber wetting, which weakened the fiber–matrix interfacial bonding and hindered effective stress transfer at the interface.

Japins et al. [20] fabricated polypropylene-based composites incorporating Cordenka fibers, HM400X cellulose microfibers, and their hybrid combinations at varying fiber contents, examining their tensile and flexural properties. Their results showed that incorporating both Cordenka fibers and cellulose microfibers into the polypropylene matrix increased the tensile and flexural strengths by 34% and 21%, respectively, at approximately 27 vol% reinforcement. The maximum tensile and flexural strengths of the Cordenka fiber composites were observed to be 45% and 28% higher, respectively, than those of the HM400X cellulose microfiber composites. Furthermore, the fracture strain of the hybrid composite was impressive, closely matching that of the Cordenka composite and other conventional materials. They concluded that the hybrid composites exhibited superior mechanical performance compared to wood–plastic composites (WPCs) and certain engineering plastics like polyamides.

The reviewed literature indicates that reinforcing thermoplastic and thermosetting matrices like PP, PLA, and EP with Cordenka fibers has significantly enhanced their mechanical performance. However, further research is needed to optimize the processing and manufacturing techniques for regenerated cellulose fiber-reinforced PP composites to fully realize improvements in performance metrics such as tensile strength, modulus, and impact resistance. Consequently, this study introduces a novel approach for fabricating continuous Cordenka/PP composite strands using the Multi-Pin-assisted Resin Infiltration (M-PaRI) method [21,22], followed by injection molding of the cut strands to produce test specimens. Since the M-PaRI method produces strands consisting of continuous yarns, it has the advantage of obtaining long pellets for injection molding. The mechanical and morphological properties of the resultant composites were then compared with those of PP matrix injection-molded composites reinforced with ramie, a typical cellulosic natural fiber that also yields continuous strands due to its twisted yarn structure.

## 2. Experimental Methods

### 2.1. Test Materials

**Figure 1:**
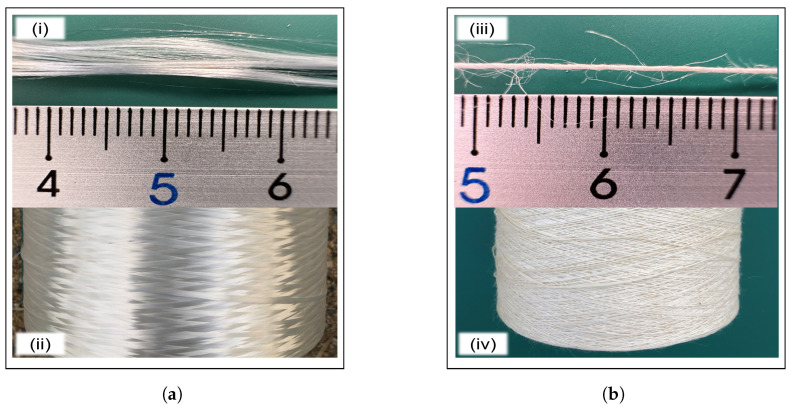
Reinforcement raw materials: (**a**) Cordenka single yarn, showing (i) the yarn itself and (ii) its spool, and (**b**) ramie single twisted yarn, showing (iii) the yarn itself and (iv) its spool. The scale applies exclusively to the yarns.

Cordenka single yarn regenerated fiber (Cordenka 700, 1840 dtex, f 1000; supplied by GmbH & Co. KG, Obernburg, Germany) was utilized as the reinforcement material in this study. On the other hand, ramie single twisted yarn (No. 16; a typical natural cellulosic fiber manufactured by TOSCO Co., Ltd., Tokyo, Japan) served as the comparative material. Figure 1 shows these raw materials, and Table 2 outlines their physical properties [22,23]. Additionally, polypropylene (PP J-900GP, $M_{W}=35\times 10^{4}$, $T_{m}=180$ °C, Idemitsu Petrochemical Co., Ltd., Tokuyama, Japan) was used as the base material [24,25], while maleic anhydride-grafted polypropylene (MAPP, Kayaku Akzo Co., Ltd., Tokyo, Japan) was added as a compatibilizer at approximately 2 wt% relative to the base material. MAPP was preferred for its excellent compatibility not only with natural cellulose such as wood fiber, but also with regenerated cellulose like Cordenka [26].

### 2.2. Single Fiber Tensile Test

**Figure 2:**
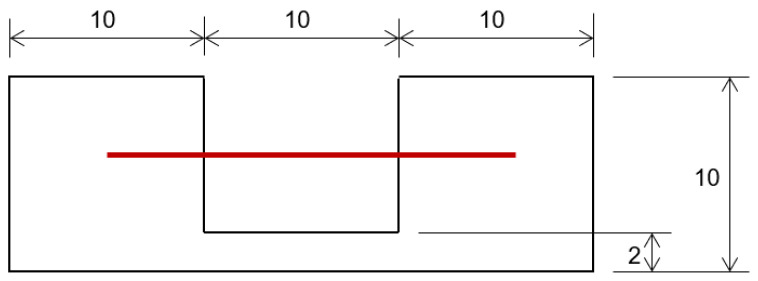
Schematic diagram illustrating the setup for tensile testing of Cordenka and ramie monofilaments (indicated by the bold red line) using gripper paper with a grid pattern, akin to graph paper. The gripper paper securely grips the ends of the monofilaments during testing, ensuring accurate measurement of their tensile properties.

Single fiber tensile specimens were prepared according to the test method for single fibers specified in JIS standards (JIS R7606: 2000). Initially, gripper paper was cut to the dimensions and shape depicted in Figure 2. Cordenka or ramie single fibers were then positioned on top of the paper as indicated by the bold solid red line and secured using cellophane tape and instant adhesive. The gauge length of each specimen was set to 10 mm.

The single fiber tensile tests were conducted using a micro load cell tensile compression testing machine (manufactured by Yamaguchi University). First, a video microscope (KH-1300, HIROX Co., Ltd., Tokyo, Japan) was used to measure the diameters of the single fibers in two directions: arbitrary and orthogonal, and then the cross-sectional areas of the single fibers were calculated from the measured data [27]. The measured arbitrary and orthogonal diameters were designated as the major and minor axes, respectively, and the cross-sectional area was estimated by approximating the fiber cross-section to an ellipse. Measurements were taken at 50 points per specimen, and the average value of the elliptical cross-sectional area was taken as the representative cross-sectional area. Tensile tests were conducted after cutting the thin middle part of the paper tab immediately before the test. The tests were performed at room temperature and cross-head speed of 1 mm/min to ensure consistency in strength. To account for variability in strength, the number of tests conducted was set to at least 20 specimens for each condition.

### 2.3. Continuous Strand Molding Method

Cordenka and ramie composite strands were developed using the M-PaRI method [21]. This method utilized a single-axis extruder which combined the coating and resin impregnation processes. The distinctive feature of this method is its ability to develop composite strands without pre-cutting individual strands. Generally, the mechanical properties of short fiber-reinforced composites depend on the fiber length, and cutting individual strands typically leads to decreased mechanical performance. In conventional processing techniques, achieving optimal fiber dispersion while maintaining fiber length during the kneading process is inherently difficult, often resulting in the prioritization of dispersion for quality control purposes. However, the method utilized in this study offers a distinct advantage, enabling the production of GC materials without severing individual fibers. This approach ensures uniform fiber dispersion while preserving their original lengths, thereby enhancing the overall material properties.

**Figure 3:**
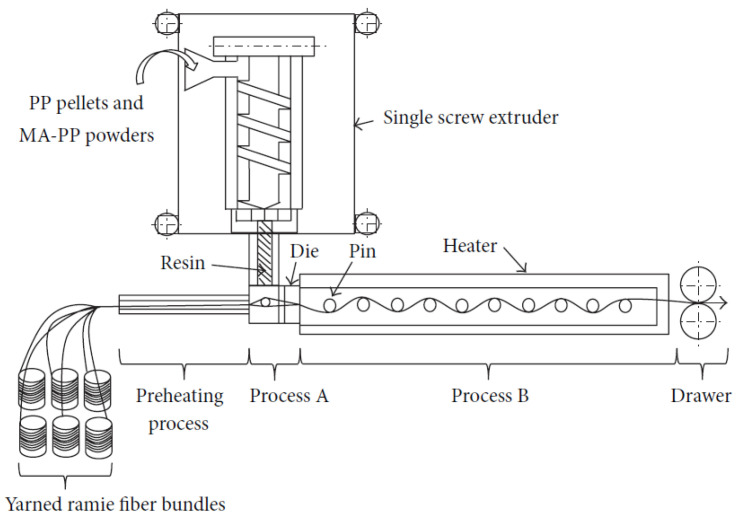
Schematic illustration of the Multi-pin-assisted resin infiltration (M-PaRI) technique [21].

The single-axis extruder, equipped with a 15 mm spinning device, was used in this study (Musashino Kikai Co., Ltd., Tokyo, Japan). Using this system, PP blended with MAPP was kneaded at a barrel zone temperature of 180 °C and a screw speed of 5.0 r.p.m. This extruder offers key advantages, such as precise and consistent control over melt temperature and pressure at the coating die section, along with uniform kneading of the PP. A schematic of the strand continuous forming method, M-PaRI, utilized in this study is shown in Figure 3.

**Figure 4:**
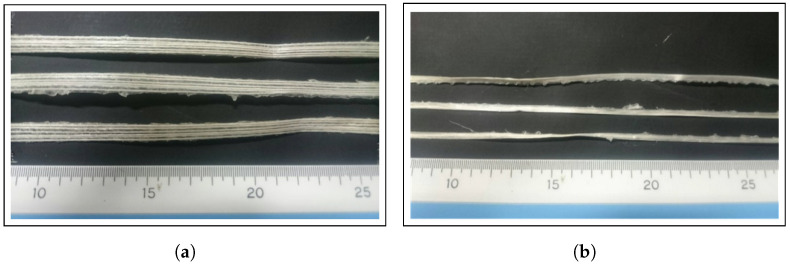
(**a**) Ramie and (**b**) Cordenka yarn composite strands prepared by combined-PaRI technique.

The kneaded PP resin was coated onto preheated ramie spun yarn (single yarn) as it passed through the die in Process A. Subsequently, in Process B, the yarn was pulled through multiple pins (37 pins) placed inside a heater, alternately coming into contact with the pins, which allowed the resin to impregnate the fibers within the single yarn. The molded ramie and Cordenka yarn composite strands shown in Figure 4 were then drawn out through the die at a speed of 800 rpm.

### 2.4. Preparation of Tensile and Impact Test Specimens

**Figure 5:**
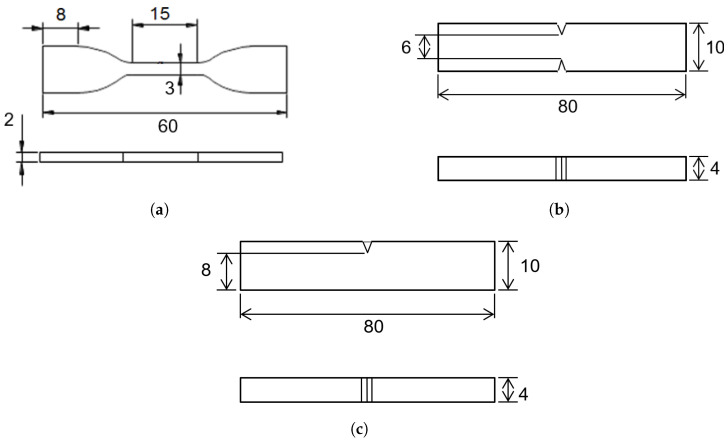
CAD geometric models of (**a**) ASTM D638 dumbbell-shaped tensile test specimen, (**b**) JIS 7111 tensile test specimen with a notch on either side, and (**c**) JIS 7111 impact test specimen with a notch on one side.

In order to further improve fiber dispersion, the fabricated ramie and Cordenka strands were cut into pellets approximately 15 mm in length and kneaded using a biaxial compounding machine (No.655 Laboplast Mill). The fiber content was determined to be 30 wt% by comparing the prepared pellets with individual yarns. To adjust the fiber content to 20 wt% and 15 wt%, PP and MAPP were added to the pellets in ratios of 0.67:1 and 1:1, respectively. The cut pellets, along with the added materials, were then kneaded at a cylinder temperature of 190 °C, with a screw rotation speed of 30 rpm, and for a mixing time of 10 min. Subsequently, the compounded materials were ground into pellets approximately 5 mm in length using a grinder (P-1314, Horai Co., Ltd., Osaka, Japan). Babyplast (6/10P) injection molding machine (Rambaldi + Co. srl, San Giovanni in Persiceto, Italy) was utilized to produce dumbbell-shaped and strip-shaped specimens at a molding temperature of 200 °C. The dumbbell-shaped specimens were designed with dimensions of 15 mm gauge length, 3 mm width, and 2 mm thickness, in accordance with ASTM D638 standards. The strip-shaped specimens measured 10 mm in width, 4 mm in thickness, and 80 mm in length, following JIS 7111 standards. Additionally, for the strip-shaped specimens, variants were created with no notches, notches on one side, and notches on both sides. The notch dimensions were specified as a depth of 2 mm, a radius of 0.25 mm, and an angle of 45°. Figure 5 illustrates the geometric models and dimensions (in mm) of the specimens used in this study.

### 2.5. Tensile Test

Tensile tests were performed on both dumbbell-shaped and strip-shaped specimens using a compact tabletop tensile testing machine (LSC-1/30D, 1 kN capacity, manufactured by JT Tosi). Prior to testing, the thickness and width of each specimen were measured with a caliper, and the corresponding cross-sectional areas were calculated. Subsequently, tensile tests were conducted at room temperature and crosshead speed of 10 mm/min. Five specimens were tested in each experimental run, and data on test duration, applied load, and strain were recorded using a data logger, amplifier, and strain gauges (KGF-5-120-C1-11, Kyowa Electronic Instruments Co., Ltd., Tokyo, Japan). The strain gauges were positioned at mid-span on one side of each specimen, attached with an adhesive for strain gauges at one end, while the other ends were connected to the strain amplification device.

### 2.6. Fracture Surface Observation

After the tests, fracture surfaces were observed using a field emission scanning electron microscope (JSM-7500F, JEOL Ltd., Akishima, Tokyo, Japan). The observations were conducted at a distance of 15 mm from each fracture location on the specimens. For this study, one dumbbell-shaped specimen and two rectangular specimens were selected from each composition used in the tensile and impact tests: 15 wt% ramie/PP, 20 wt% ramie/PP, 15 wt% Cordenka/PP, and 20 wt% Cordenka/PP. Great care was taken to ensure that the selected fracture surfaces were oriented horizontally during the selection process.

### 2.7. Fiber Length Measurement

The fiber lengths in the dumbbell-shaped specimens were determined using a Soxhlet extractor (Water Bath BS600, Yamato Scientific Co., Ltd., Tokyo, Japan). This apparatus allows for the extraction of insoluble components, such as ramie or Cordenka fibers, from the solid matrix by dissolving the polymer component, in this case PP, using an appropriate solvent. Subsequently, the extracted fibers were dispersed on a slide glass and observed for fiber length using a video microscope. The Soxhlet tests were conducted on one dumbbell-shaped specimen each for 15 wt% ramie/PP, 20 wt% ramie/PP, 15 wt% Cordenka/PP, and 20 wt% Cordenka/PP, with the lengths of 500 fibers observed and recorded as test results.

### 2.8. Impact Test

Charpy impact testing was carried out using an Izod–Charpy impact testing machine (CIT-25JCI). The specimens tested included rectangular specimens both with and without notches. Prior to testing, the thickness, width, and length of each specimen were measured using calipers to determine the cross-sectional area. The impact tests were performed using a 2J hammer weighing 0.857 kg, with a 2.80 cm distance between the centers. Each experimental run consisted of testing five specimens, and all tests were conducted at room temperature.

## 3. Results and Discussion

### 3.1. Single Fiber Tensile Test Results

**Figure 6:**
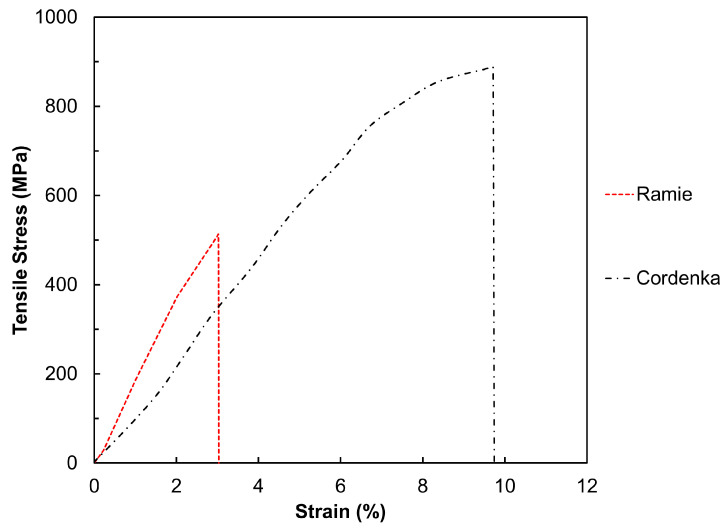
Typical stress–strain curves of Cordenka and ramie monofilaments.

Table 3 shows the results of the single fiber tensile tests for Cordenka and ramie fibers. The coefficients of variation are indicated in parentheses, and the corresponding representative stress–strain curves are shown in Figure 6. Strains were calculated based on the applied crosshead speeds and the specimen gauge length shown in Figure 1. The results indicate that although Cordenka fibers had a lower Young’s modulus than ramie fibers, they exhibited superior tensile strength and fracture strain. The tensile strengths of some cellulosic natural fibers, such as flax [28] and curaua [29], have been reported to compare favorably with Cordenka. However, their fracture strain is significantly lower than that of Cordenka, typically ranging from 2 to 4% [30,31]. Thus, despite the similarity in tensile strength, Cordenka retains the advantage of higher fracture strain. In terms of fracture energy, or toughness, Cordenka fibers exhibited values more than three times higher. Furthermore, it is worth noting that Cordenka fibers consistently showed lower coefficients of variation compared to ramie fibers, corroborating more stable properties.

### 3.2. Tensile Test Results of Dumbbell-Shaped Neat PP, Cordenka/PP, and Ramie/PP Specimens

**Figure 7:**
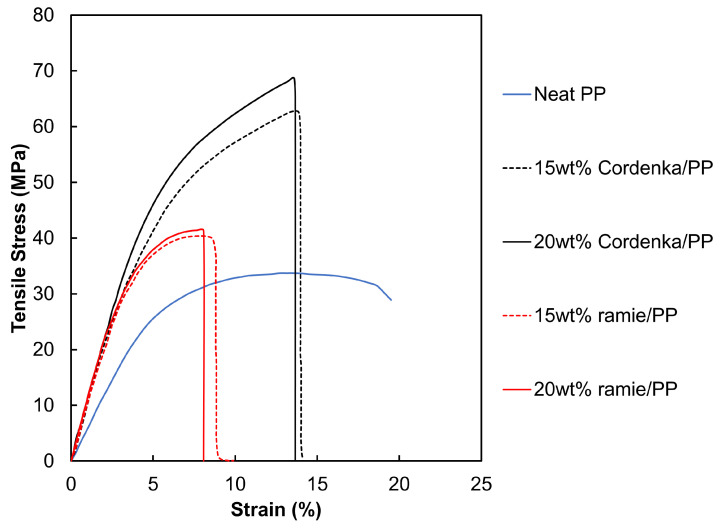
Typical stress–strain curves of neat PP, Cordenka/PP and ramie/PP composites.

Tensile test results of neat PP, Cordenka/PP and ramie/PP specimens are presented in Table 4, with the representative stress–strain diagrams shown in Figure 7. The stress–strain curves clearly indicate that both Cordenka/PP and ramie/PP composites exhibited higher tensile strengths than the neat PP, with the 20 wt% Cordenka/PP composite yielding the highest tensile strength of 68.7 MPa. Additionally, tensile strength further increased with increase in fiber content. Specifically, at 20 wt% fiber content, the tensile strength of ramie/PP and Cordenka/PP composites increased by 22.6% and 105%, respectively, compared to neat PP. In a similar study involving synthetic fibers, Chen et al. [32] reinforced neat PP with aramid fiber (AF) and reported a maximum tensile strength of 67.8 MPa at 20 wt% AF, representing a 111.8% improvement over neat polypropylene.

Fernandez et al. [33] also reinforced the PP matrix with recycled carbon fiber (rCF) and achieved maximum tensile strength exceeding 60 MPa with 30 wt% rCF. Both authors reported a decrease in fracture strain with an increase in fiber content. Ari et al. [34] analyzed the mechanical performance of polypropylene composites reinforced with chopped glass fiber (GF), carbon fiber (CF), and aramid fiber (AF). They found that the most significant tensile strength improvements occurred at 30 vol.% fiber content for each material. GF reinforcement increased the tensile strength from 38.0 MPa to 78.5 MPa, CF increased it to 74.2 MPa, and AF raised it to 72.2 MPa. From the above comparative studies, it may be infered that the tensile properties of Cordenka/PP composites achieved in this current study show comparable performance to traditional synthetic fiber-reinforced PP composites.

In absolute terms, both the 15 wt% and 20 wt% Cordenka/PP composites showed approximately 1.6 times higher tensile strength than the ramie/PP composites. This finding aligns with the properties of the single Cordenka and ramie fibers used in this study, illustrated earlier in Figure 6, where a single Cordenka fiber exibited about 1.5 times higher tensile strength than a single ramie fiber. Also, as described in Section 3.1, the Young’s modulus of Cordenka monofilament was observed to be lower than that of ramie monofilament. However, in the reinforced composites, this situation is reversed, with the Young’s modulus of Cordenka/PP composites reported to be slightly higher than that of ramie/PP composites.

Furthermore, to assess the magnitude of strength improvement on PP matrix achieved by the Cordenka fibers used in this study, findings from previous literature on the strength increase observed with CNF fiber-reinforced PP matrices—referred to in the introduction as ’processed’ fibers—are summarized and compared with the current results. For instance, Kahavita et al. [8] reported a maximum tensile strength of 27.8 MPa in silane surface-modified CNF/PP composites at 3.5 wt% CNF, representing a 12.6% increase over neat PP. Similarly, Norrrahim et al. [35] observed a tensile strength increase of at least 31% in neat PP after incorporating 3 wt% CNF filler. In another study, they found that the optimal tensile strength was achieved with CNF concentrations of 1% to 3% [36]. Based on this comparison, it is evident that the performance of the Cordenka/PP composites developed in this study exceeded that of CNF-reinforced PP composites reported in previous studies.

In addition to the PP matrix, polylactic acid (PLA) has also been extensively studied, with many researchers attempting to utilize the impressive strength properties of CNF reinforcement in a PLA matrix. This focus has been driven by the fact that PLA is a biodegradable resin, hence CNF/PLA is a fully green composite, i.e., more environmentally friendly compared to composites with natural fiber reinforcements compounded with recyclable PP matrix. For example, Jonoobi et al. [37] obtained maximum tensile strength of 71 MPa, and reported a 22.4% and 24.1% increase in the tensile and flexural strengths, respectively, of PLA upon reinforcement with 5 wt% CNF. Senkum et al. [38] melt-compounded poly(methyl methacrylate)-functionalized CNFs in PLA matrix and achieved a tensile strength of 79 MPa, an improvement of nearly 30% over neat PLA at 20 wt% reinforcement loading. Other researchers pointed out that this strength increase is due to the nucleation effect of CNFs on the matrix [39,40]. In cellulose nanowhisker (CNW)- and cellulose nanocrystal (CNC)-reinforced PLA nanocomposites, it has also been reported that CNW and CNC induce crystal nucleation of PLA and increase its crystallinity [41,42,43]. It has further been confirmed that such functioning as a crystal nucleus can also occur through nanofibrils formed by fibrillation of ramie fibers, although the resin is polyamide [44]. Since PP is also a semi-crystalline thermoplastic, the reinforcing effect of CNFs described above is considered to be crystallization strengthening of the matrix material rather than fiber reinforcement.

On the other hand, other researchers have reported a decline in tensile strength of thermoplastic polymer materials as a result of adding CNF, even though in some cases other mechanical properties were enhanced. For instance, Yang et al. [7] reported a maximum tensile strength of 21.3 MPa with 2 wt% CNF filler in a PP matrix, which was actually a 3.0% decrease from the original strength of neat PP. Although Young’s modulus consistently improved with increasing CNF content from 0 wt% to 10 wt%, the tensile strength results were less favorable. Similarly, Jung et al. [6] found that CNF/PP composites developed from N_2_ plasma-treated PP and alkaline CNF achieved a maximum tensile strength of 24.5 MPa at 1 wt% CNF, reflecting a 27.9% decline from the tensile strength of neat PP. Zhang et al. [45] also experimented with 0.5 wt% CNF in the PLA matrix and noticed an 18.4% increase in tensile modulus, whereas the tensile strength dropped by 7.4%, from 47.5 to 44 MPa. They attributed this drop in tensile strength to poor interfacial adhesion between CNF and PLA.

Thus, from the sampled literature discussed above, it may be pointed out that the mechanical properties of thermoplastic composites reinforced with Cordenka and ramie fibers compare favorably with those of CNF fiber-reinforced thermoplastic composites. However, higher concentrations of Cordenka and ramie fibers than CNF fibers are necessary for the realization of these comparable properties. This is because the optimum fiber content for the fiber-reinforcing effect is much larger than that of crystallization strengthening, enabling it to far exceed the maximum possible strength that can be achieved by the matrix. On the other hand, the reported decline in mechanical properties of CNF/PP composites resulting from an increase in CNF content beyond optimum values, typically between 1 wt% and 5 wt%, is due to agglomeration of CNF, which is a well-known and widely reported defect in CNF-based polymer composites [46,47,48,49,50,51,52]. To solve this problem, research on chemical modification of CNF is currently being conducted with intent to improve nanodispersibility of CNF in polymer matrices [53,54,55,56,57]. In any case, high fiber content can be achieved in Cordenka and ramie fiber-reinforced polymer composites due to low fiber-to-fiber cohesion. This is because macroscale fibers have a significantly smaller specific surface area compared to nanoscale CNFs.

In the fracture strain results presented in Figure 7, the tensile tests for the neat polypropylene (PP) specimens were terminated at 20% strain, as the material exhibited continued elongation without attaining fracture. On the other hand, the fracture strains of the Cordenka/PP composites were found to be greater than those of the ramie/PP composites. While both composites showed similar initial slopes in the elastic region, the Cordenka/PP composites exhibited significant strain hardening in the nonlinear region, indicating a larger area under the stress–strain curve, which implies higher fracture energy. Looking at the fracture energy data shown in Table 4 obtained by numerical approximation of the area under each corresponding stress–strain curve, it can be seen that Cordenka/PP composites showed fracture energy values approximately 2.1 to 2.5 times higher than those of ramie/PP composites. Furthermore, as evidenced by Figure 7, the nonlinear region of both composite materials tended to align with the strain level where the PP matrix entered its nonlinear region, suggesting that the onset of nonlinearity was influenced by the deformation behavior of the matrix material. This behavior contrasted markedly with that observed in the Cordenka and ramie monofilament tensile stress–strain curves, where Cordenka fibers exhibited significant deformation resistance before failure, unlike ramie fibers. Therefore, the high strain hardening observed in the nonlinear region of the monofilament test results was likely dependent on fiber behavior. As discussed later, ramie fibers did not reach a critical fiber length within the matrix material, resulting in ramie/PP composites exhibiting behavior dominated by the deformation of the PP matrix even in the nonlinear region.

### 3.3. Observation of Fracture Surfaces

**Figure 8:**
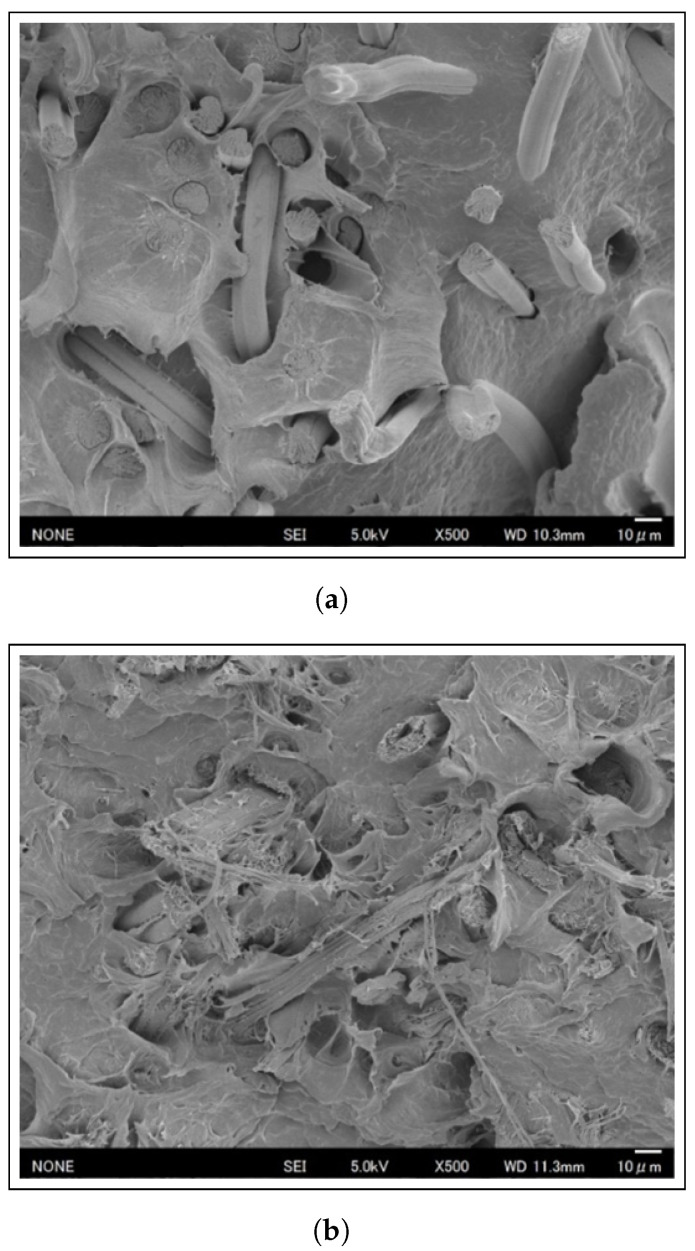
SEM fracture surface images of (**a**) 20 wt% Cordenka/PP (**b**) 20 wt% ramie/PP specimen.

Figure 8 shows representative SEM images of fracture surfaces of 20 wt% Cordenka/PP and 20 wt% ramie/PP composites. Looking at the fracture surface of the Cordenka/PP composite in Figure 8a, there is clear evidence of weak fiber–matrix adhesion, indicated by extensive fiber pull-out. The SEM images further reveal fiber breakage accompanied with clear gaps in the PP matrix, with some loose fibers visibly protruding from the fracture surface. These gaps and the presence of pulled-out fibers suggest a lack of effective stress transfer between the matrix and the reinforcing fibers, likely due to insufficient interfacial bonding. These findings contrast with those of Chen et al. [32], who, through SEM and Polarized Optical Microscopy (POM) analysis of the fractured surface of AF/PP composites, confirmed strong fiber–matrix interfacial adhesion. The combination of fiber pull-out and breakage points to a mixed-mode failure, where adhesion between the fibers and the matrix is insufficient to prevent debonding under load, while the fibers themselves are unable to sustain higher stress levels. This highlights the need for enhanced surface treatment of fibers or optimized matrix–fiber interactions to improve the overall mechanical performance of Cordenka/PP composites. Thus, to achieve better adhesion, further investigation into suitable coupling agents is necessary. However, based on the results of Soxhlet tests discussed later, Cordenka fibers remained longer in the matrix compared to ramie fibers. This retention of fibers in the matrix prevented crack propagation during the pull-out process, hence the observed exhibition of high fracture energy [58].

Conversely, a careful examination of the scanning electron microscope image of the fracture surface of the ramie/PP composite reveals minimal fiber pull-out and few gaps between the fibers and the resin, indicating good adhesive strength (see Figure 8b). The adhesion between natural fibers, recycled cellulose fibers, and the PP/MAPP matrix is influenced by the surface roughness of the fibers. Smooth surfaces, such as those of Cordenka fibers, are at a disadvantage in this regard [59]. Based on these observations, it can be inferred that ramie/PP composites exhibit superior adhesion compared to Cordenka/PP composites under the MAPP to PP ratio utilized in this study.

### 3.4. Fiber Length Measurement Results

**Figure 9:**
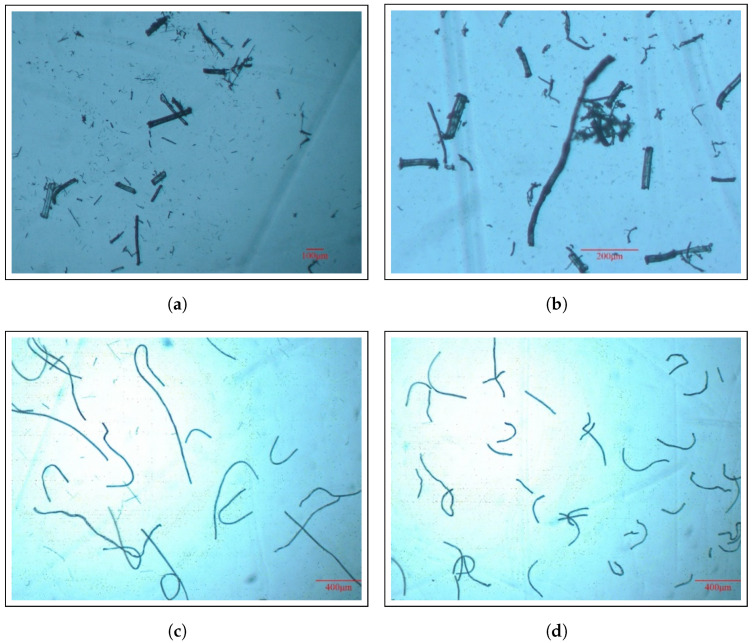
Soxhlet test images showing the state of fiber dispersion on the surfaces of (**a**) 15 wt% ramie/PP, (**b**) 20 wt% ramie/PP, (**c**), 15 wt% Cordenka/PP, and (**d**) 20 wt% Cordenka/PP composites.

Figure 9 shows the appearance of the fibers after the Soxhlet test. It is clearly evident from the images that the fibers in the Cordenka/PP composite, especially in the 15 wt% sample, remained relatively long. Also, from the fiber length measurement results presented in Table 5, it is evident that in the 20 wt% sample, the fiber lengths decreased to about half their initial lengths. This suggests that as the fiber content increases, fibers are more likely to entangle and break during the kneading process. On the other hand, for the ramie/PP composites, many relatively short fibers were observed in both the 15 wt% and 20 wt% samples. The aspect ratio of these fibers was found to be between six and eight, indicating that the fiber reinforcing effect was insufficient to significantly enhance the mechanical properties of the composite.

**Figure 10:**
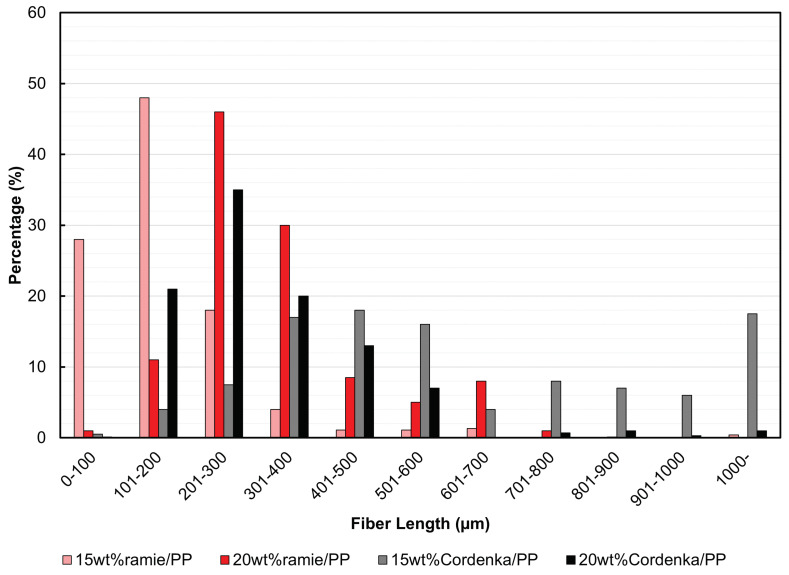
Fiber length distribution graph.

To quantitatively assess this effect, the critical fiber length $l_{c}$ was calculated using the following equation, based on the Kelly–Tyson model [60].

(1) $$l_{c}=\frac{d_{f}}{2\tau_{my}}\cdot \sigma_{f}^{*}$$

where $d_{f}$ is the fiber diameter or width, $\sigma_{f}^{*}$ is the fiber strength, and $\tau_{my}$ is the shear yield stress of the matrix resin. The shear yield stress $\tau_{my}$ of the PP matrix resin was determined using fracture strain levels observed during the composites’ tensile testing, which closely approached PP’s tensile strength. This value was adjusted according to the Mises criterion by dividing it by $\sqrt{3}$. It was found that the average length of the extracted Cordenka fibers exceeded the calculated $l_{c}$, whereas ramie fibers remained below $l_{c}$. Furthermore, from the frequency distribution graph of the measured fiber lengths shown in Figure 10, it is evident that many of the Cordenka fibers in the 15 wt% composite and about half of those in the 20 wt% composite exceeded $l_{c}$. On the other hand, many of the ramie fibers fell below $l_{c}$. Although the Young’s modulus of ramie fiber is higher than that of Cordenka fiber, this difference in length likely causes the reversal of Young’s modulus between the two composites, as shown in Table 4. However, the observed variations in fiber length do not appear to have a significant effect on the increase in tensile strength. This is evident when comparing the strength ratio of Cordenka to ramie (C/r), calculated from Table 3 (1.56), with the ratios of the composites in Table 4, which are 1.55 for 15%C/r and 1.66 for 20%C/r.

### 3.5. Tensile Test Results of Notched Strip Neat PP, Cordenka/PP, and Ramie/PP Specimens

**Figure 11:**
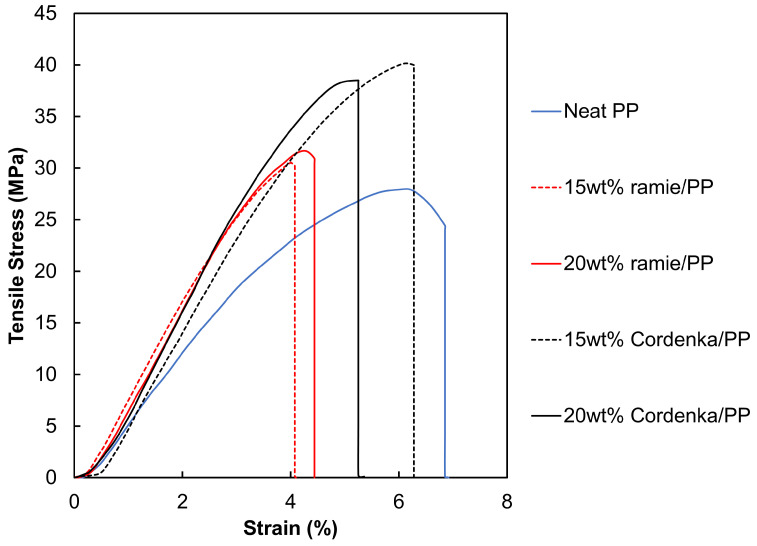
Typical stress–strain curves of notched tensile specimens.

The resistance of Cordenka/PP composites to notches was evaluated by comparing the properties of notched specimens to unnotched ones. Table 6 presents the tensile test results of strip specimens with notches on both sides and their ratios compared to dumbbell-shaped (unnotched) specimens, and Figure 11 illustrates the stress–strain curves for the notched test specimens. The results indicate that even with the presence of notches, the tensile strength of Cordenka/PP composites still exceeded that of both ramie/PP and neat PP composites. Furthermore, the Cordenka/PP composites exhibited higher fracture energy compared to ramie/PP composites and achieved levels comparable to neat PP. Notably, the 15 wt% Cordenka/PP composite displayed the highest tensile strength and fracture energy among all the tested materials.

Focusing on the tensile strength and fracture energy ratio to the unnotched dumbbell-shaped specimens, it is evident that Cordenka/PP composites exhibited greater reduction compared to other composites, indicating that they do not exhibit very good resistance to notches. Comparing Figure 11, which illustrates stress–strain curves of notched specimens, with Figure 7 showing the stress–strain curve of the dumbbell-shaped specimen discussed before, it is evident that a significant portion of the nonlinear region is lost. In other words, stress concentration due to the notches hinders strain hardening in the nonlinear region. This implies that the stress concentration causes the Cordenka fibers to break prematurely, significantly reducing tensile strength and fracture energy. On the other hand, a similar tendency of significant loss in the nonlinear region is observed in ramie/PP composites. This is because, as mentioned earlier, the ramie/PP do not have excellent strain-hardening properties. Therefore, it deduced that ramie/PP composites do not experience a significant a decrease in strength and energy compared to Cordenka/PP composites.

### 3.6. Impact Test Results

Table 7 shows the results of the Charpy impact test. For the PP specimens without notches, the impact test was performed using a 2.0 J hammer under the same conditions as the other tests. However, since the specimens did not break, the impact values obtained using a 7.5 J hammer were recorded. These results showed that the Cordenka/PP specimens exhibited higher impact strength than the ramie/PP specimens, with the 15 wt% Cordenka/PP specimens achieving the highest impact strength of 47.4 kJ/m^2^. More specifically, the impact energy of Cordenka/PP composites was approximately 2 to 2.5 times greater than that of the ramie/PP specimens, irrespective of the presence of notches. This value is nearly comparable to the multiple of fracture energy observed in the dumbbell-shaped tensile test shown in Table 2. Furthermore, it significantly exceeds the ratios shown in the notched tensile test results in Table 4. This suggests that Cordenka/PP composites are superior in absorbing impact energy compared to ramie/PP composites.

In the study by Chen et al. [32], the Izod notched impact strength of AF-reinforced PP composites peaked at 40.1 kJ/m^2^ with 40 wt% AF loading. Similarly, Fernandez et al. [33] reported a maximum impact strength of over 35 kJ/m^2^ for rCF/PP composites at 20 wt% rCF. Both studies on non-biodegradable fibers reported impact strength values lower than those observed for Cordenka/PP composites in the current work, supporting the hypothesis that Cordenka/PP composites can competitively match the mechanical performance of traditional synthetic fiber-reinforced plastic composites.

Additionally, in the case of Cordenka/PP composites, it has been reported that adding non-compatibilizing agents [61] or omitting compatibilizers entirely [26] slightly reduces static strength but significantly enhances impact strength. Generally, in composite materials with low interfacial strength, crack propagation tends to be dispersed through interfacial debonding, which allows for greater energy absorption. However, low interfacial strength also results in slower stress recovery from the fiber ends, which is detrimental to achieving high static strength. Consequently, Cordenka/PP composites exhibit a higher impact energy ratio compared to the tensile strength ratio of ramie/PP composites.

As for the neat PP specimens, large variations in impact strength values were observed in the notched specimens, indicating that PP material is highly susceptible to the effects of notches. Focusing on the specimens without notches, it can be seen that as the fiber content decreased, the impact strength of both Cordenka/PP and ramie/PP specimens increased. On the other hand, for specimens with notches, an increase in fiber content led to an increase in impact strength. These trends are likely due to the inherent weakness of neat PP against notches. The elongation of the fibers and their pullout from the matrix are believed to effectively absorb impact energy and mitigate crack propagation originating from the notch.

## 4. Conclusions

This study examined the mechanical and morphological properties of polypropylene composites reinforced with Cordenka fibers, comparing them with those reinforced with ramie fibers. The composite strands were produced using the M-PaRI continuous forming method, cut and injection molded into dumbbell- and strip-shaped specimens with varying fiber content, and subjected to tensile and impact testing. SEM observation results revealed that the fractured surface of Cordenka/PP specimens was characterized by pulled-out fibers with gaps and broken fibers at the interface, indicating a mixed-mode failure. In contrast, the ramie/PP composite fracture surfaces showed minimal fiber pull-out and less scale gaps. Fiber length analysis showed that Cordenka fibers retained lengths exceeding the critical threshold within the matrix. Cordenka/PP composites also exhibited significantly higher tensile strength and toughness than ramie/PP composites. For instance, maximum tensile strength of 68.7 MPa was achieved by the 20 wt% Cordenka/PP dumbbell PP specimens, while the corresponding ramie/PP specimens exhibited a maximum tensile strength of 41.4 MPa at the same fiber loading. Additionally, Cordenka/PP composites showed higher fracture energy values, approximately 2.1 to 2.5 times greater than those of the ramie/PP composites. In the notched tensile test, it was deduced that stress concentration at the notch prevented strain hardening in the nonlinear region. This concentration caused the Cordenka fibers to break prematurely, leading to a significant reduction in both tensile strength and fracture energy.

The impact test results of unnotched fiber-reinforced specimens indicated that the 15 wt% Cordenka/PP specimens exhibited the highest impact energy of 47.7 kJ/m^2^ compared to 19.6 kJ/m^2^ for the ramie/PP specimens. It was inferred that composites with low interfacial strength exhibited crack propagation predominantly through interfacial debonding, facilitating enhanced energy absorption. However, this low interfacial strength also impeded stress recovery at the fiber ends, which adversely affects the attainment of high static strength. Consequently, Cordenka/PP composites demonstrated a higher impact energy ratio compared to the tensile strength ratio observed in ramie/PP composites. In conclusion, the regenerated fibers utilized in this study exhibited exceptional performance, comparable to that of natural fibers, indicating their potential to replace synthetic fibers.

Future research should focus on optimizing the composite manufacturing processes utilized in this study to achieve mechanical properties that surpass those currently attained. Additionally, recognizing the inherent weakness of neat polypropylene against notches, which resulted in lower toughness of Cordenka/PP specimens compared to the established toughness of Cordenka fibers, it is recommended that future studies explore the use of Cordenka fiber-reinforced toughened polypropylene or other thermoplastic resins. Such studies should aim to determine whether the toughness of notched composite specimens can be enhanced through the incorporation of these alternative matrix materials.

## Figures and Tables

**Table 1:** Mechanical properties of representative thermoplastic resins.

Mechanical Property	Resin Type			
	PP	PE	PVC	PS
Specific Gravity	0.90–0.91	0.94–0.97	1.30–1.58	1.04–1.09
Young’s Modulus (MPa)	1098–1548	412–1235	2401–4116	2746–4120
Fracture Strain (%)	200–700	20–1300	40–80	1–6
Tensile Strength (MPa)	29.4–38.3	20.6–38.3	41.2–52.0	34.3–82.4
Compressive Strength (MPa)	38.3–55.0	18.6–24.5	54.9–89.2	79.4–109.8
Bending Strength (MPa)	41.2–55.0	33.3–48.1	68.7–109.8	54.9–96.1
Izod Impact Strength (J/m)	11–117	27–1068	21–1068	13–21

**Table 2:** Physical properties of Cordenka and ramie fibers.

Fiber Type	Density (g/cm^3^)	Width ^1^/Diameter (µm)	Length (µm)	Moisture Content (%)
Ramie [22]	1.5	40	150 ^2^	8
Cordenka 700, 1840 dtex, f 1000 [23]	1.5	12.5	Continuous	13

^1^ The cross-section of ramie fiber cells is not circular, so the lateral dimension is referred to as width. ^2^ The value shown represents the average fiber length.

**Table 3:** Tensile properties of single ramie and Cordenka fibers obtained in the current study.

Fiber Type	No. of Specimens	Tensile Strength (MPa)	Young’s Modulus (GPa)	Fracture Strain (%)	Fracture Energy (MJ/m^3^)
Cordenka	23	888 (0.19)	7.5 (0.29)	12.0 (0.21)	1.07 (0.34)
ramie	22	571 (0.31)	20.5 (0.56)	3.30 (0.36)	0.30 (0.51)

**Table 4:** Tensile test results of dumbbell-shaped neat PP, ramie/PP and Cordenka/PP specimens.

Specimen Type	Number of Samples	Tensile Strength (MPa)	Young’s Modulus ^1^ (GPa)	Fracture Energy (MJ/m^3^)
Neat PP	5	33.7	1.84	-
15 wt% Cordenka/PP	5	62.6	3.16	6.68
20 wt% Cordenka/PP	5	68.7	3.24	5.85
15 wt% ramie/PP	5	40.3	2.74	2.64
20 wt% ramie/PP	5	41.4	2.90	2.82

^1^ Calculated from strains measured by strain gauge.

**Table 5:** Soxhlet fiber length results for each specimen.

Specimen Type	Avg. Fiber Length [µm]	Aspect Ratio	Crit. Fiber Length [µm]	Fiber Diameter [µm]
15 wt% ramie/PP	158	6	369	25
20 wt% ramie/PP	210	8		
15 wt% Cordenka/PP	650	52	298	13
20 wt% Cordenka/PP	330	26		

**Table 6:** Tensile test results of strip specimens with notches in comparison to dumbbell specimens.

Specimen Type	Tensile Strength [MPa]	Strength Ratio to Unnotched	Fracture Energy [MJ/m^3^]	Energy Ratio to Unnotched
Neat PP	28.40	0.84	1.28	–
15 wt% ramie/PP	30.28	0.75	0.83	0.314
20 wt% ramie/PP	31.38	0.75	0.72	0.255
15 wt% Cordenka/PP	40.15	0.64	1.52	0.228
20 wt% Cordenka/PP	38.50	0.56	1.10	0.188

**Table 7:** Charpy impact energies of Cordenka/PP and Ramie/PP composites.

Specimen Type	Impact Energy [kJ/m^2^]	Impact Energy [kJ/m^2^]	Energy Ratio
	Unnotched (U)	Notched (N)	(N/U)
Neat PP	66.8 (7.5J)	1.8	-
15 wt% ramie/PP	19.6	3.7	0.189
20 wt% ramie/PP	19.5	3.9	0.200
15 wt% Cordenka/PP	47.7	8.7	0.182
20 wt% Cordenka/PP	40.5	10.0	0.247

## Data Availability

The data that support the findings of this study are available from the corresponding author upon request.

## Abbreviations

The following abbreviations are used in this manuscript:

AF	Aramid Fiber
ASTM	American Society for Testing and Materials
CF	Carbon Fiber
rCF	Recycled Carbon Fiber
CNF	Cellulose Nanofiber
CNC	Cellulose Nanocrystal
CNW	Cellulose Nanowhisker
EP	Epoxy
GC	Green Composite
GF	Glass Fiber
JIS	Japanese Industrial Standards
MAPP	Maleic Anhydride Grafted Polypropylene
M-PaRI	Multi-Pin-Assisted Resin infiltration
MTM	Medium Temperature Molding
PLA	Polylactic Acid
PE	Polyethylene
PP	Polypropylene
PS	Polystyrene
PVC	Polyvinyl Chloride
POM	Polarized Optical Microscopy
SEM	Scanning Electron Microscopy
WF	Wood Flour
WP	Wood-Polypropylene Composite

## References

[1] Cakmak O.K. (2024). Biodegradable polymers—A review on properties, processing, and degradation mechanism. Circ. Econ. Sustain..

[2] Paul S., Sen B., Das S., Abbas S.J., Pradhan S.N., Sen K., Ali S.I. (2023). Incarnation of bioplastics: Recuperation of plastic pollution. Int. J. Environ. Anal. Chem..

[3] Japan Society of Mechanical Engineers (1977). Mechanical Engineering Handbook.

[4] Gross S., Tarragano G., MacBride R.R., Port C.O. (1973). Modern Plastics Encyclopedia.

[5] Vaidya U.K., Chawla K. (2008). Processing of fibre reinforced thermoplastic composites. Int. Mater. Rev..

[6] Jung B.N., Jung H.W., Kang D., Kim G.H., Lee M., Hwang S.W., Shim J.K. (2020). The fabrication of affinity improved nanocomposites with plasma treated polypropylene (PP) and alkaline cellulose nanofiber (CNF) suspension. Polym. Test..

[7] Yang H.S., Gardner D.J. (2011). Mechanical properties of cellulose nanofibril-filled polypropylene composites. Wood and Fiber Sci..

[8] Kahavita K., Samarasekara A., Amarasinghe D., Karunanayake L. Influence of surface modification of cellulose nanofibers (CNF) as the reinforcement of polypropylene based composite. Proceedings of the 2019 Moratuwa Engineering Research Conference (MERCon).

[9] Niihara K.i., Noguchi T., Makise T., Kashima W., Endo M., Isogai A. (2022). Cellulose nanofibril/polypropylene composites prepared under elastic kneading conditions. Cellulose.

[10] Aoki K. (2024). Development of Cellulose Nanofiber (CNF)/PP Composites. Oleo Sci..

[11] Yang Z., Li X., Si J., Cui Z., Peng K. (2019). Morphological, mechanical and thermal properties of poly (lactic acid)(PLA)/cellulose nanofibrils (CNF) composites nanofiber for tissue engineering. J. Wuhan Univ. Technol.-Mater. Sci. Ed..

[12] Yan X., Shao Y., Gao Z., Wu B., Wang C., Zhu C., Tu L. (2024). Preparation and properties of cellulose nanofiber/Ti3C2Tx/Poly (lactic acid) composite film. J. Polym. Res..

[13] Agnes E.A., Hillig E., Zattera A.J., Beltrami L.R., Covas J.A., Hilliou L., Sousa J.D., Calado L., Pinto M., de Andrade Lucas A. (2024). Potentialities of cellulose nanofibers (CNFs) in low density polyethylene (LDPE) composites. Eur. J. Wood Wood Prod..

[14] Norrrahim M.N.F., Kasim N.A.M., Knight V.F., Halim N.A., Shah N.A.A., Noor S.A.M., Jamal S.H., Ong K.K., Yunus W.M.Z.W., Farid M.A.A. (2021). Performance evaluation of cellulose nanofiber reinforced polymer composites. Funct. Compos. Struct..

[15] Bax B., Müssig J. (2008). Impact and tensile properties of PLA/Cordenka and PLA/flax composites. Compos. Sci. Technol..

[16] Ganster J., Fink H.P., Pinnow M. (2006). High-tenacity man-made cellulose fibre reinforced thermoplastics–injection moulding compounds with polypropylene and alternative matrices. Compos. Part A Appl. Sci. Manuf..

[17] Kovalovs A., Kalnins K., Franciszczak P., Bledzki A. Low velocity impact response of polypropylene biocomposites reinforced with man-made cellulose and soft wood fibres. Proceedings of the 20th International Scientific Conference Engineering for Rural Development.

[18] Meredith J., Coles S.R., Powe R., Collings E., Cozien-Cazuc S., Weager B., Müssig J., Kirwan K. (2013). On the static and dynamic properties of flax and Cordenka epoxy composites. Compos. Sci. Technol..

[19] Malaba T., Wang J. (2015). Unidirectional Cordenka Fibre-Reinforced Furan Resin Full Biocomposite: Properties and Influence of High Fibre Mass Fraction. J. Compos..

[20] Japins G., Franciszczak P., Kalnins K., Kovalovs A. (2019). Mechanical Properties of Polypropylene Biocomposites Reinforced with Man-Made Cellulose Fibres and Cellulose Microfibres. Proc. IOP Conf. Ser. Mater. Sci. Eng..

[21] Kim H.B., Goda K., Noda J., Aoki K. (2013). Developing simple production of continuous ramie single yarn reinforced composite strands. Adv. Mech. Eng..

[22] Kim H.B., Goda K. (2020). Production of a single ramie spun yarn/PP composite tape and reliability analysis in elastic modulus. Seikei-Kakou.

[23] Rayon C. (2022). Technical Data Sheet Multifilament Yarn.

[24] Sato S., Oka K., Murakami A. (2004). Heat transfer behavior of melting polymers in laminar flow field. Polym. Eng. Sci..

[25] Sato S., Sakata Y., Aoki J., Kubota K. (2006). Effects of filler on heat transmission behavior of flowing melt polymer composites. Polym. Eng. Sci..

[26] Franciszczak P., Bledzki A. (2016). Tailoring of dual-interface in high tenacity PP composites–Toughening with positive hybrid effect. Compos. Part A Appl. Sci. Manuf..

[27] Tanabe K., Matsuo T., Goda K., Ohki J. (2008). Strength Evaluation of Kurawa Fibers Considering Cross-Sectional Area Variation. Materials.

[28] Baley C., Bourmaud A., Davies P. (2021). Eighty years of composites reinforced by flax fibres: A historical review. Compos. Part A Appl. Sci. Manuf..

[29] Gomes A., Matsuo T., Goda K., Ohgi J. (2007). Development and effect of alkali treatment on tensile properties of curaua fiber green composites. Compos. Part A Appl. Sci. Manuf..

[30] Jörg M. (2010). Industrial Applications of Natural Fibres: Structure, Properties and Technical Applications.

[31] Mohanty A.K., Misra M., Drzal L.T. (2005). Natural Fibers, Biopolymers, and Biocomposites.

[32] Chen X., Zhang S., Xu G., Zhu X., Liu W. (2012). Mechanical, flammability, and crystallization behavior of polypropylene composites reinforced by aramid fibers. J. Appl. Polym. Sci..

[33] Fernández A., Santangelo-Muro M., Fernández-Blázquez J.P., Lopes C.S., Molina-Aldareguia J.M. (2021). Processing and properties of long recycled-carbon-fibre reinforced polypropylene. Compos. Part B Eng..

[34] Ari A., Bayram A., Karahan M., Karagöz S. (2022). Comparison of the mechanical properties of chopped glass, carbon, and aramid fiber reinforced polypropylene. Polym. Polym. Compos..

[35] Norrrahim M.N.F., Ariffin H., Yasim-Anuar T.A.T., Hassan M.A., Ibrahim N.A., Yunus W.M.Z.W., Nishida H. (2021). Performance evaluation of cellulose nanofiber with residual hemicellulose as a nanofiller in polypropylene-based nanocomposite. Polymers.

[36] Norrrahim M.N.F., Yasim-Anuar T., Jenol M., Nurazzi N.M., Sapuan S., Ilyas R. (2021). Performance evaluation of cellulose nanofiber reinforced polypropylene biocomposites for automotive applications. Biocomposite and Synthetic Composites for Automotive Applications.

[37] Jonoobi M., Harun J., Mathew A.P., Oksman K. (2010). Mechanical properties of cellulose nanofiber (CNF) reinforced polylactic acid (PLA) prepared by twin screw extrusion. Compos. Sci. Technol..

[38] Senkum H., Kelly P.V., Ahmad A.A., Es-haghi S.S., Gramlich W.M. (2024). Strengthening polylactic acid (PLA) composites with poly (methyl methacrylate)-functionalized cellulose nanofibrils created through grafting-through emulsion polymerization. RSC Appl. Polym..

[39] Ambone T., Torris A., Shanmuganathan K. (2020). Enhancing the mechanical properties of 3D printed polylactic acid using nanocellulose. Polym. Eng. Sci..

[40] Frone A.N., Berlioz S., Chailan J.F., Panaitescu D.M. (2013). Morphology and thermal properties of PLA–cellulose nanofibers composites. Carbohydr. Polym..

[41] Sullivan E.M., Moon R.J., Kalaitzidou K. (2015). Processing and characterization of cellulose nanocrystals/polylactic acid nanocomposite films. Materials.

[42] Sanchez-Garcia M.D., Lagaron J.M. (2010). On the use of plant cellulose nanowhiskers to enhance the barrier properties of polylactic acid. Cellulose.

[43] Sung S.H., Chang Y., Han J. (2017). Development of polylactic acid nanocomposite films reinforced with cellulose nanocrystals derived from coffee silverskin. Carbohydr. Polym..

[44] Matsunaga T., Sato Y., Ozaki T., Macadre A., Goda K. (2021). Crystallization reinforcement of biobased PA resin by fibrillation of natural fibers. J. Jpn. Soc. Compos. Mater..

[45] Zhang Z., Cao B., Jiang N. (2023). The mechanical properties and degradation behavior of 3D-printed cellulose nanofiber/polylactic acid composites. Materials.

[46] Hwang S., Han Y., Gardner D.J. (2024). Morphological characteristics of spray dried cellulose nanofibers produced using various wood pulp feedstocks and their effects on polypropylene composite properties. Compos. Part B Eng..

[47] Pandey J.K., Nakagaito A.N., Takagi H. (2013). Fabrication and applications of cellulose nanoparticle-based polymer composites. Polym. Eng. Sci..

[48] Yasim-Anuar T.A.T., Ariffin H., Norrrahim M.N.F., Hassan M.A., Tsukegi T., Nishida H. (2019). Sustainable one-pot process for the production of cellulose nanofiber and polyethylene/cellulose nanofiber composites. J. Clean. Prod..

[49] Yasim-Anuar T.A.T., Ariffin H., Norrrahim M.N.F., Hassan M.A., Andou Y., Tsukegi T., Nishida H. (2020). Well-dispersed cellulose nanofiber in low density polyethylene nanocomposite by liquid-assisted extrusion. Polymers.

[50] Keeratipinit K., Wijaranakul P., Wanmolee W., Hararak B. (2024). Preparation of High-Toughness Cellulose Nanofiber/Polylactic Acid Bionanocomposite Films via Gel-like Cellulose Nanofibers. ACS Omega.

[51] Mehdinia M., Farajollah Pour M., Yousefi H., Dorieh A., Lamanna A.J., Fini E. (2024). Developing Bio-Nano Composites Using Cellulose-Nanofiber-Reinforced Epoxy. J. Compos. Sci..

[52] Matsumoto K., Takemura K., Kitamura R., Katogi H., Tanaka T., Takagi H. (2024). Cellulose nanofiber-introduced continuous-ramie yarn-reinforced polylactic acid filament for 3D printing: Novel fabrication process and mechanical properties. Compos. Part A Appl. Sci. Manuf..

[53] Yamato K., Yoshida Y., Kumamoto Y., Isogai A. (2022). Surface modification of TEMPO-oxidized cellulose nanofibers, and properties of their acrylate and epoxy resin composite films. Cellulose.

[54] Liu S., Low Z.X., Xie Z., Wang H. (2021). TEMPO-oxidized cellulose nanofibers: A renewable nanomaterial for environmental and energy applications. Adv. Mater. Technol..

[55] Ning R., Ono Y., Isogai A. (2024). Effects of UV irradiation of TEMPO-oxidized cellulose nanofibril/water dispersions on chemical structure, molar mass, and morphology. Cellulose.

[56] Goi Y., Fujisawa S., Saito T., Yamane K., Kuroda K., Isogai A. (2019). Dual functions of tempo-oxidized cellulose nanofibers in oil-in-water emulsions: A pickering emulsifier and a unique dispersion stabilizer. Langmuir.

[57] Qu J., Yuan Z., Wang C., Wang A., Liu X., Wei B., Wen Y. (2019). Enhancing the redispersibility of TEMPO-mediated oxidized cellulose nanofibrils in N, N-dimethylformamide by modification with cetyltrimethylammonium bromide. Cellulose.

[58] Matthews F.L., Rawlings R.D. (1999). Composite Materials: Engineering and Science.

[59] Graupner N., Rößler J., Ziegmann G., Müssig J. (2014). Fibre/matrix adhesion of cellulose fibres in PLA, PP and MAPP: A critical review of pull-out test, microbond test and single fibre fragmentation test results. Compos. Part A Appl. Sci. Manuf..

[60] Hull D. (1981). An Introduction to Composite Materials.

[61] Feldmann M. (2016). The effects of the injection moulding temperature on the mechanical properties and morphology of polypropylene man-made cellulose fibre composites. Compos. Part A Appl. Sci. Manuf..

